# Ansichten von Lehrbeauftragten zur digitalen Transformation in der HNO-Lehre: Aktueller Stand und Perspektiven in Aus- und Weiterbildung

**DOI:** 10.1007/s00106-024-01468-1

**Published:** 2024-04-08

**Authors:** T. Hildenbrand, S. Kuhn, F. Everad, F. Hassepaß, M. Neudert, C. Offergeld

**Affiliations:** 1 Klinik für Hals‑, Nasen- und Ohrenheilkunde, Universitätsklinikum Freiburg, Killianstr. 5, 79106 Freiburg, Deutschland; 2 Institut für Digitale Medizin, Universitätsklinikum Gießen-Marburg & Philipps-Universität Marburg, Marburg, Deutschland; 3 Klinik für Hals-Nasen- und Ohrenheilkunde, Universitätsklinikum Carl-Gustav-Carus, Dresden, Deutschland

**Keywords:** Digitalisierung im Gesundheitswesen, Curriculare Lehre, Ärztliche Weiterbildung, Digitale Technologie, Digitale Lehrmethoden, Digital health, Curricular teaching, Advanced training, Digital technology, Digital teaching formats

## Abstract

**Hintergrund:**

Die digitale Transformation in der curricularen Lehre des Medizinstudiums beinhaltet zum einen die zunehmende Nutzung digitaler Lehr‑/Lernformate und zum anderen die Vermittlung von digitalen ärztlichen Kompetenzen. Auch in der fachärztlichen Weiterbildung müssen im Zuge der Veränderungen Konzepte der Wissensvermittlung und Kompetenzprofile überdacht und vermittelt werden.

**Ziel der Arbeit:**

Ziel der vorliegenden Studie war die Ermittlung des aktuellen Stands der digitalen Transformation in der HNO-Lehre in Aus- und Weiterbildung an universitären Hals‑, Nasen- und Ohrenkliniken.

**Material und Methoden:**

Ein Fragebogen mit 9 Fragen zu Themen der digitalen Transformation wurde an die Lehrbeauftragten der 37 nationalen universitären Hals‑, Nasen- und Ohrenkliniken verschickt. Die Umfrage erfolgte online anonym über das Umfrageportal SurveyMonkey® (San Mateo, CA, USA).

**Ergebnisse:**

An der Umfrage nahmen 86,5 % der angeschriebenen Lehrbeauftragten teil. Nur 25 % der HNO-Kliniken bieten eine Lehrveranstaltung zur Vermittlung digitaler Kompetenzen für Studierende an. Digitale Lehrmethoden kommen nur in der Hälfte der Kliniken zum Einsatz. Nur 56,25 % der Lehrbeauftragten erhalten bei der Umsetzung der digitalen Transformation Unterstützung. In 40,62 % der Kliniken wird die digitale Transformation in der Weiterbildung thematisiert, aber nur 28,12 % der teilnehmenden Kliniken wenden digitale Lehrmethoden in der fachärztlichen Weiterbildung an.

**Schlussfolgerung:**

Insbesondere im Bereich der curricularen Lehre werden einige Aspekte der digitalen Transformation bereits umgesetzt, nicht zuletzt auch getrieben durch die COVID-19-Pandemie. Insgesamt zeigt sich allerdings noch ein deutlicher Nachholbedarf sowohl in der Ausbildung von Studierenden als insbesondere auch in der Ausbildung von Weiterbildungsassistenten der HNO-Heilkunde.

## Abstract (en)

**Background:**

Digital transformation in curricular teaching in medicine comprises the use of digital teaching and learning formats as well as the transfer of digital skills for medical staff. Concepts of knowledge transfer and competency profiles also have to be adapted and transferred in advanced training due to necessary changes.

**Objective:**

The aim of this study was an evaluation of the current state of digital transformation in otorhinolaryngology teaching in undergraduate and advanced training at otorhinolaryngology departments of university medical centers in Germany.

**Materials and methods:**

A questionnaire with nine questions on digital transformation was sent to the assistant professors of 37 national university ENT departments. The anonymous survey was conducted online via the online platform SurveyMonkey®.

**Results:**

Of the contacted assistant professors, 86.5% participated in the survey. Teaching sessions on digital skills for medical students are part of the curriculum in only 25% of ENT departments. Digital teaching formats are used by half of the departments in undergraduate training. Only 56.25% of the assistant professors receive support to realize the changes required by digital transformation. In 40.62% of departments, the issue of digital transformation is broached during advanced training, but only 28.12% use digital teaching methods to train junior doctors.

**Conclusion:**

Aspects of digital transformation are implemented mainly in undergraduate education, partly driven by the COVID 19 pandemic. Overall, there is still considerable backlog in undergraduate and advanced training in ENT.

## Digitalisierung in der curricularen Lehre und Weiterbildung

Die digitale Transformation im Gesundheitswesen soll schnellere und effizientere Abläufe schaffen und die Behandlungsqualität von Patienten verbessern, z. B. durch eine sichere und ständige Verfügbarkeit von Patientendaten und durch die Möglichkeit der Auswertung großer Datenmengen durch künstliche Intelligenz (KI) und den Einsatz von „clinical decision support systems“. Auch die Kommunikation zwischen Arzt und Patient und somit die Arzt-Patienten-Beziehung wird sich in Zukunft durch die Digitalisierung weiter verändern. Um zukünftige Ärztinnen und Ärzte für dieses sich ständig ändernde Umfeld vorzubereiten, ist es nötig, bereits im Studium und in der Weiterbildung Aspekte der digitalen Transformation zu thematisieren.

Sprechen wir von digitaler Transformation in der curricularen Lehre, so sind 2 Aspekte zu berücksichtigen. Der erste Themenkomplex ist die Nutzung der Digitalisierung in der didaktischen Gestaltung. Dies beinhaltet digitale Lernangebote, Blended Learning, den Einsatz von KI und Apps sowie Virtual-Reality-Trainingsprogramme. Die Digitalisierung des Lehrangebots ermöglicht den Studierenden ein Lernen unabhängig von Raum und Zeit. Die COVID-19-Pandemie hat die Digitalisierung im Medizinstudium gezwungenermaßen deutlich vorangetrieben. Sowohl für die Vermittlung theoretischer als auch für praktische Lehrinhalte wurden digitale Lösungen gefunden und umgesetzt. Nach Ende der Pandemie besteht allerdings die Gefahr, dass erzielte Fortschritte wieder in den Hintergrund geraten.

Der zweite Aspekt sind Lehrangebote zu Themen der Digitalisierung, wie eHealth und Telemedizin, digitale Gesundheitsanwendungen, Big Data, elektronische Patientenakte, KI, Augmented/Virtual/Mixed Reality (AR/VR/MR) und ethische, rechtliche und ethische Aspekte dieser Transformation. Entsprechende Lehrinhalte waren 2015, in der ersten Fassung des Nationalen Kompentenzbasierten Lernzielkatalogs Medizin (NKLM), gar nicht und in der Fassung von 2021 vergleichsweise begrenzt abgebildet. Der aktuelle Referentenentwurf der Ärztlichen Approbationsordnung bildet sowohl die Digitalisierung in didaktischer als auch curricularer Hinsicht umfassend ab [1]. Die Umsetzung wird jedoch nach einer erneuten Verschiebung voraussichtlich erst 2027 verpflichtend erfolgen [2].

Beide Aspekte gelten prinzipiell sowohl für die fachärztliche Weiterbildung als auch für die ärztliche Fortbildung.

Im Oktober 2023 fand das erste nationale HNO-Lehrbeauftragtentreffen in Freiburg statt. Eines der behandelten und diskutierten Themen war die digitale Transformation in der Lehre. Um den aktuellen Stand der digitalen Transformation in der HNO-Lehre und -Weiterbildung zur evaluieren, führten die Autoren eine Umfrage bei allen Lehrbeauftragten universitärer HNO-Kliniken durch.

## Studiendesign und Untersuchungsmethoden

### Studienablauf

**Figure 1:**
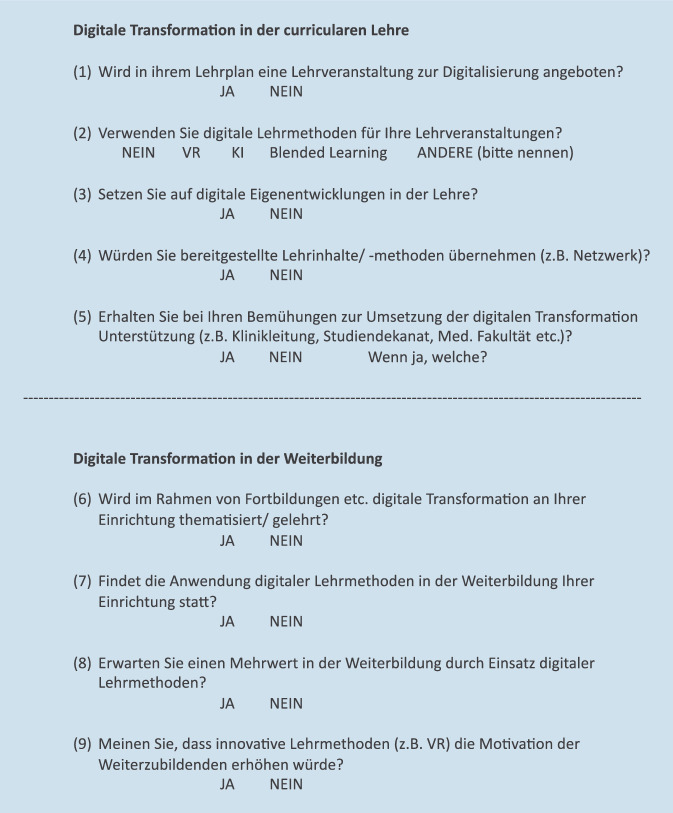

Ein Fragebogen mit 9 Fragen zu Themen der digitalen Transformation in der curricularen Lehre und Weiterbildung der HNO wurde erstellt und in das Umfrageportal SurveyMonkey® eingepflegt (Abb. 1).

Über den Verteiler der Arbeitsgruppe Lehren und Prüfen in der HNO-Heilkunde der Deutschen Gesellschaft für Hals-Nasen-Ohren-Heilkunde, Kopf- und Hals-Chirurgie e. V. (DGHNO-KHC) wurden alle Lehrbeauftragten angeschrieben und um Teilnahme an der Umfrage gebeten. Die Umfrage erfolgte online anonym über das Umfrageportal SurveyMonkey®.

Die Daten wurden anonym in Excel gespeichert und ausgewertet. Die Studienleiter erhielten die anonymen Daten zur Auswertung. Es wurden keine personenbezogenen Daten erhoben, gespeichert oder ausgewertet. Die Studienleiter hatten zu keinem Zeitpunkt Zugang zu persönlichen und personenbezogenen Daten der Teilnehmer. Eine Identifikation der Teilnehmenden war nicht möglich.

### Ethische Gesichtspunkte

Die Studie wurde im Einklang mit nationalem Recht und der Deklaration von Helsinki in ihrer aktuellen Fassung durchgeführt. Die Daten wurden anonym erhoben, ein Rückschluss auf die Teilnehmer war nicht möglich, weshalb nach Rücksprache mit der zuständigen Ethikkommission der Albert-Ludwigs-Universität Freiburg keine Genehmigung der Studie durch jene erforderlich war.

## Ergebnisse

Insgesamt wurden 37 Lehrbeauftragte angeschrieben und um Teilnahme gebeten. Es wurden 32 Fragebögen beantwortet. Dies entspricht einer Rücklaufquote von 86,5 %.

### Digitale Transformation in der curricularen Lehre

**Figure 2:**
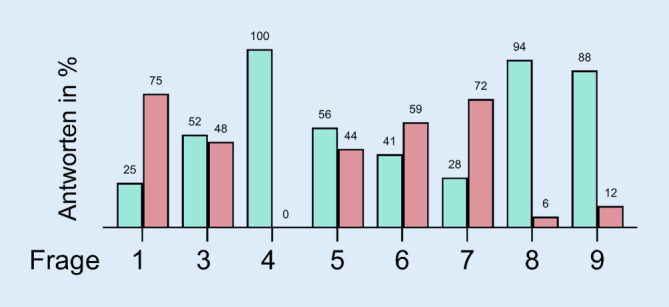

**Figure 3:**
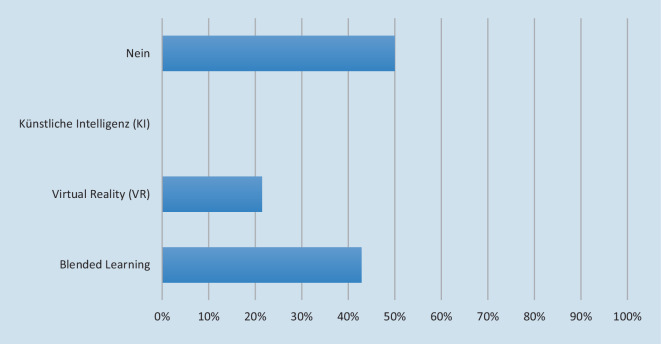

Die prozentuale Verteilung der Antworten auf die Fragen 1 und 3–9 ist in Abb. 2 dargestellt. Auf die Frage, ob im Lehrplan der jeweiligen Abteilung eine Lehrveranstaltung zur Digitalisierung angeboten wird, antworteten 25 % mit ja und 75 % mit nein. Bei der Frage, ob und, wenn ja, welche digitalen Lehrmethoden für Lehrveranstaltungen verwendet werden, gaben 50 % der Teilnehmenden an, dass sie keine entsprechenden Lehrmethoden anwenden. Von den Teilnehmenden, die mit ja antworteten, verwenden 21,43 % Virtual Reality und 42,86 % Blended Learning (Abb. 3). In den Freitextantworten wurden zudem Live und 3‑D-Übertragungen von Operationen, App-basierte TED-Abstimmungen (Tele-Dialog), videobasierte Selbstkontrollen, Simulatoren und E‑Learning-Angebote angegeben. Auf digitale Eigenentwicklungen in der Lehre setzen 51,64 %. Alle Teilnehmenden gaben an, dass sie bereitgestellte Lehrinhalte bzw. Lehrmethoden, z. B. im Rahmen eines Netzwerks, übernehmen würden. Und 56,25 % gaben an, dass sie bei der Umsetzung der digitalen Transformation Unterstützung erhalten. Die Freitextantworten nach der Art der Unterstützung sind in Tab. 1 zusammengefasst.

**Table 1:** 

Es wurden Videos gezeigt, wie man z. B. eine Vorlesung vertonen kann
EDV
Klinikleitung und Dekanat
Lehrstuhl Medizindidaktik und Studiendekanat: Content Production, Digitalisierung, Webspace
Konkrete Hilfestellung durch Beauftragte des Dekanats und Lehrgänge werden angeboten
Finanzielle Unterstützung
Fakultät, Team der IT, Lehre
Ressourcen Klinikleitung
Von der Medizindidaktik werden verschiedene Workshops angeboten
Anschaffung von Touchpads für Digitalisierungskurs durch Klinikleitung/Lehrleitung, Angebot der Zusammenarbeit mit Lehrklinik: Virtual Reality, Einbettung des Digitalisierungskurses im Rahmen der Vorlesungsreihe „Digitalisierung in der Medizin“
Unterstützung durch Klinikleitung und Unterstützung durch Studiendekanat soll etabliert werden
Finanziell nach entsprechender Ausschreibung, keine bei der konkreten Realisierung

### Digitale Transformation in der Weiterbildung

Bei der HNO-ärztlichen Weiterbildung wird in 40,62 % der Abteilungen digitale Transformation, z. B. im Rahmen von Fortbildungen, gelehrt oder thematisiert. Digitale Lehrmethoden werden in der Weiterbildung nur von 28,12 % der Einrichtungen angewendet. Einen Mehrwert in der Weiterbildung durch den Einsatz digitaler Lehrmethoden erwarten 93,75 % der Teilnehmenden. Zudem sind 87,5 % der Auffassung, dass innovative Lehrmethoden die Motivation der Weiterzubildenden erhöhen würde.

## Diskussion

Ziel der vorliegenden Studie war die Evaluation des Stands der digitalen Transformation in der curricularen Lehre und Weiterbildung in der Hals‑, Nasen‑, Ohrenheilkunde an deutschen Universitätskliniken. Hierzu befragten die Autoren bundesweit die Lehrbeauftragten der universitären HNO-Kliniken. Aufgrund der sehr guten Rücklaufquote von 86,5 % gehen sie von einem repräsentativen Ergebnis aus. Insgesamt zeigt sich, dass bereits einige Aspekte der digitalen Transformation umgesetzt wurden. Gleichzeitig zeigt sich aber noch ein sehr deutliches Verbesserungspotenzial.

### Lehrangebote zur Digitalisierung in der curricularen Lehre

Lehrangebote zur Digitalisierung werden nur in 25 % der universitären Einrichtungen in der HNO-Heilkunde angeboten. Der NKLM sieht verschiedene Kompetenzen im Bereich der Digitalisierung in der Medizin vor. Wie und wo diese umgesetzt werden sollen, bleibt jedoch offen [3]. Es wird das Wissen und die Bewertung von Themen wie digitale Dokumentation, elektronische Patientenakte, Fehlermöglichkeiten und Risiken von IT-Systemen, KI, Telemedizin und Datenschutzaspekte, digitale Arzt-Patienten-Kommunikation, Mobile Health, Data Literacy, AR und VR gefordert. Gerade die angestrebte Vermittlung von Fertigkeiten in, Haltungen zu und damit einhergehend die Bewertung dieser Themen setzt tiefergehende Kenntnisse sowohl aufseiten der Lehrenden als auch aufseiten der Lernenden voraus. Auch für die HNO-Heilkunde sind diese Themen relevant und sollten in das Curriculum aufgenommen werden. In einer Schweizer Studie wurden die Studiengangverantwortlichen der Dekanate der 7 Schweizer Universitäten und der Eidgenössischen Technischen Hochschule (ETH) Zürich zu Lehrangeboten zur Vermittlung digitaler Kompetenzen befragt [4]. Auch wenn alle Befragten die zu vermittelnden Themen der Digitalisierung als relevant einstuften, zeigte sich ein heterogenes Bild, was die Umsetzung betrifft. Nur 2 der Universitäten gaben an, einen speziellen Kurs zu Themen der Digitalisierung anzubieten. In den übrigen Universitäten werden die Inhalte fächerübergreifend über die Dauer des Studiums verteilt angeboten. Als Hemmnisse für die Etablierung von Kursangeboten wurden mangelnde Unterstützung in der Entwicklung und Durchführung, mangelndes Personal und Budget und fehlende Inhalte aufgeführt.

### Anwendung digitaler Lehrmethoden

Nur die Hälfte der Teilnehmenden gab an, dass sie digitale Lehrmethoden anwenden. Digitale Lehrmethoden bieten im Gegensatz zu klassischen Lehrmethoden verschiedene Vorteile. Digitale Lehrinhalte erlauben ein zeitlich und räumlich unabhängiges Lernen. Die Studierenden können in ihrem eigenen Tempo lernen und Inhalte beliebig oft wiederholen. Dies stellt auch in der Weiterbildung einen erheblichen Vorteil dar. Beim Konzept des Blended Learning und Flipped Classroom werden Lerninhalte von den Studierenden in einer selbstgesteuerten Lernphase anhand von zur Verfügung gestellten Lernmaterialien vorbereitet und anschließend in Präsenzveranstaltungen angewendet und so durch aktives Lernen vertieft. Dies ermöglicht eine tiefere Verarbeitung des Erlernten. Studien konnten die Effektivität des Flipped Classroom und die gute Akzeptanz vonseiten der Studierenden belegen [5–10]. Jedoch zeigte eine aktuelle Studie, dass Studierende die zunehmende Digitalisierung von Lerninhalten auch kritisch beurteilen [11]. Hier wurde von den Studierenden v. a. die digitale Vermittlung praktischer Kenntnisse kritisch gesehen und der möglichst weitreichende Einsatz digitaler Lehrmethoden von etwas mehr als der Hälfte der Studierenden abgelehnt. Etwa die Hälfte der Studierenden gab zudem an, dass die Effektivität, Konzentrationsfähigkeit, Lernmotivation und Teilnahmewahrscheinlichkeit an Offline-Veranstaltungen größer sei.

Virtual Reality (VR) hat sich in den letzten Jahren immens weiterentwickelt und sowohl im privaten Bereich als auch in der Lehre vermehrt Einzug gehalten. Diese innovative Methode wird in der Lehre und Weiterbildung hauptsächlich für Simulationen eingesetzt. Simulationstraining ist v. a. für das Training von Notfall- und im klinischen Alltag seltenen Situationen (z. B. Koniotomie) sinnvoll. Es ermöglicht die Ausbildung ohne Gefährdung von Patienten. Nachteile der traditionellen Simulation mit Dummys, Simulatoren und tierischen oder humanen Präparaten sind mitunter hohe Kosten, ethische Gesichtspunkte, ein teilweise großer Platzbedarf und eine ggf. eingeschränkte Verfügbarkeit und Zugangsmöglichkeiten. Diese Einschränkungen können z. T. durch den Einsatz von VR-Simulationen umgangen werden. VR-Simulationen können außerdem einfacher adaptiert, geändert oder erweitert werden. In Studien zeigen sich allerdings unterschiedliche Ergebnisse bezüglich der Verbesserung der Kenntnisse bei einem direkten Vergleich zwischen VR und traditioneller Simulation. Insgesamt zeigten die Studienteilnehmer aber eine hohe Zufriedenheit mit dem Training mit VR-Simulationen [12].

Eine weitere Einsatzmöglichkeit der VR ist die Vermittlung anatomischer Kenntnisse. Insbesondere die regionalen Anatomie sowie die Neuro- und Gefäßanatomie scheinen aufgrund ihrer räumlichen Komplexität besonders geeignet zu sein. Die Vermittlung anhand zweidimensionaler Abbildungen ist schwierig. Der Zugang zu Humanpräparaten wird aufgrund von ethischen und gesetzlichen Beschränkungen schwieriger. Hier können 3‑D- und VR-Modelle eine gute Alternative sein. Die VR-Anatomie hat sich in mehreren Studien als geeignet erwiesen, um das anatomische Wissen, das räumliche Verständnis und die Visualisierungsfähigkeit von Studierenden zu verbessern [13–18].

Auch in der Weiterbildung können digitale Lehrmethoden sinnvoll eingesetzt werden. Der Alltag von Weiterbildungsassistenten lässt i. d. R. nur wenig Zeit sowohl für den Erwerb theoretischer als auch praktischer Kenntnisse. Digitale Lerninhalte sind jederzeit zugänglich und können so von den Weiterzubildenden unabhängig von festen Fortbildungszeiten und insbesondere Örtlichkeiten genutzt werden. Dies ermöglicht eine maximale Flexibilität. Jedoch werden digitale Lehrmethoden in der HNO-Weiterbildung nur von 28,12 % der universitären Einrichtungen angewendet. Andererseits erwarten 93,75 % der teilnehmenden Lehrbeauftragten einen Mehrwert durch den Einsatz digitaler Lehrmethoden. Es ist notwendig zu evaluieren, welche Hemmnisse zu dieser Diskrepanz führen, um die Verbreitung und den Einsatz digitaler Lehrmethoden in der Weiterbildung zu fördern. Eine Möglichkeit könnte die gemeinsame Nutzung von edukativen Ressourcen durch Einrichtung einer allen Lehrinteressierten zugänglichen digitalen HNO-Lehr-Plattform sein, welche den Kriterien der Open Educational Resources (OER) entspricht.

Hemmnisse der Digitalisierung in der curricularen Lehre und sicher auch in der Weiterbildung können in einem hohen personellen, zeitlichen und finanziellen Aufwand gesehen werden. Dennoch setzen 51,64 % der Befragten in der vorliegenden Umfrage auf digitale Eigenentwicklungen in der curricularen Lehre. Um Ressourcen zu schonen, könnten Netzwerke hilfreich sein, in denen Lehrinhalte geteilt werden können. Alle Teilnehmer der Umfrage gaben an, dass sie bereitgestellte Lehrinhalte übernehmen würden.

Es existieren bereits frei zugängliche Quellen für Lehrmaterialien, die sog. OER. Die UNESCO definiert OER als „Bildungsmaterialien jeder Art und in jedem Medium, die unter einer offenen Lizenz stehen.“ Inhalte, die unter einer offenen Lizenz veröffentlicht werden, können kostenlos genutzt, bearbeitet und weiterverarbeitet werden. Der Urheber legt dabei fest, welche Nutzungsrechte eingeräumt werden [19, 20]. Der Vorteil dieser OER ist die Möglichkeit der Nutzung qualitativ hochwertiger Lehrmaterialien unter Schonung eigener personeller und finanzieller Ressourcen. Als Hürden für die Nutzung von OER werden Schwierigkeiten bei der Identifikation relevanter OER, der Einbindung in bestehende Lehrkonzepte und der Beurteilung der Eignung einzelner Quellen und des akademischen Inhalts gesehen [21]. Zusätzlich ist die Evidenz für den Nutzen in der Lehre noch unzureichend. Die Bildung eines nationalen Netzwerks, in dem die Urheber der geteilten OERs bekannt sind und somit die Qualität des Inhalts besser beurteilt werden kann, könnte helfen, einige dieser Hemmnisse zu überwinden. Dieser Punkt wurde bereits für die HNO-Heilkunde durch die Arbeitsgruppe Lehren und Prüfen in der HNO-Heilkunde (ArGru LuP) der DGHNO-KHC während des 1. Lehrbeauftragtentreffens 2023 in Freiburg thematisiert und fand bei den Delegierten großen Zuspruch, sodass eine kurz- bis mittelfristige Umsetzung angestrebt wird.

Von den Befragten gaben 56,35 % an, dass sie bei der Umsetzung der digitalen Transformation Unterstützung personeller oder finanzieller Art (zumeist vom Krankenhausträger) sowie durch Fortbildungen erhalten. Dies ist zum einen erfreulich, andererseits stellt dies aber nur etwas mehr als die Hälfte der Kliniken dar, was bedeutet, dass ein großer Teil auf sich allein gestellt ist.

### Einfluss auf den ärztlichen Alltag

Durch die Digitalisierung des Gesundheitswesens wird sich die Rolle des Arztes verändern. In der Weiterbildungsordnung sind Kenntnisse zur Digitalisierung aktuell nicht gefordert. Nur 40,62 % der Teilnehmer der Umfrage geben an, dass die digitale Transformation im Rahmen der Weiterbildung thematisiert bzw. gelehrt wird. Da die Digitalisierung voranschreitet, ist es unumgänglich, dies auch im Rahmen der Weiterbildung zu thematisieren, da sie die alltägliche Arbeit verändern wird und digitale Kompetenzen eine große Rolle spielen werden. Nur durch den Erwerb digitaler Kompetenzen wird es (HNO-)Ärztinnen und -Ärzten möglich sein, diesen Veränderungsprozess mitzugehen und v. a. (aktiv) mitzugestalten [22].

Nicht nur Ärztinnen und Ärzte an universitären und nichtuniversitären Kliniken sind von dieser Problematik in der curricularen Lehre und Weiterbildung betroffen, sondern auch die niedergelassenen Kolleginnen und Kollegen werden nicht nur selbst in ihrem Alltag mit der Digitalisierung konfrontiert, sondern werden in Zukunft durch die Beteiligung in der Lehre im Zuge der geplanten Etablierung von Lehrpraxen gefordert. Die Praxen können aufgrund des auf den Inhaber zugeschnittenen Workflows nur bedingt auf ein zusätzlich gefordertes Angebot reagieren.

### Limitationen

Durch die Rücklaufquote von 86,5 % kann das Ergebnis dieser Befragung als repräsentativ für den aktuellen Stand der digitalen Transformation an den universitären HNO-Kliniken angesehen werden. Jedoch ist nicht auszuschließen, dass insbesondere diejenigen Lehrbeauftragten an der Studie teilnahmen, die besonders unzufrieden mit dem Zustand sind, sodass ein gewisser Selektionsbias nicht sicher ausgeschlossen werden kann. Insgesamt gehen die Autoren jedoch davon aus, dass diese Umfrage den Status quo der digitalen Transformation an universitären HNO-Kliniken zuverlässig abbildet.

Das Lehrbeauftragtentreffen in Freiburg sowie die konsekutive Umfrage 2023 erlauben eine wichtige Standortbestimmung für die HNO-Lehre [23]. Hier offenbart sich thematisch bereits bekannter und zu erwartender Handlungsbedarf [24].

## Fazit für die Praxis

- Die digitale Transformation spielt bereits jetzt eine gewichtige Rolle in der curricularen Lehre, wird noch an Bedeutung gewinnen und sollte daher in keinem Lehrplan fehlen.
- Grundsätzlich betrifft dies aber auch in gleichem Maße die (HNO-)ärztliche Weiterbildung.
- Das Lehrbeauftragtentreffen in Freiburg sowie die konsekutive Umfrage 2023 erlauben eine wichtige Standortbestimmung für die HNO-Lehre.
- Hier offenbart sich thematisch bereits bekannter und zu erwartender Handlungsbedarf.
- Diesbezüglich haben sich die HNO-Lehrbeauftragten bereits für den Aufbau einer in Bezug auf Open Educational Resources (OER-)konformen, nationalen digitalen Plattform ausgesprochen, welche nun kurz- bis mittelfristig initiiert werden soll.
- Planung, Umsetzung und Akzeptanz werden sicherlich wesentliche Teile des Programms bei der kommenden Lehrbeauftragtenversammlung während der Jahrestagung der Deutschen Gesellschaft für Hals‑, Nasen- und Ohren-Heilkunde, Kopf- und Hals-Chirurgie (DGHNO-KHC) 2024 in Essen einnehmen.
